# Clinical significance of monitoring hypothyroidism in patients with autoimmune rheumatic disease: a retrospective cohort study

**DOI:** 10.1038/s41598-021-93300-x

**Published:** 2021-07-05

**Authors:** Sho Fukui, Yukihiko Ikeda, Yuko Kataoka, Haruyuuki Yanaoka, Hiromichi Tamaki, Tokutarou Tsuda, Mitsumasa Kishimoto, Hiroshi Noto, Sachiko Ohde, Masato Okada

**Affiliations:** 1 Immuno-Rheumatology Center, St. Luke’s International Hospital, 9-1 Akashi-cho, Chuo-ku, Tokyo, 104-8560 Japan; 2 Department of Internal Medicine and Rheumatology, Juntendo University Urayasu Hospital, Chiba, Japan; 3 Department of Rheumatology, NTT East Japan Kanto Hospital, Tokyo, Japan; 4 Department of Nephrology and Rheumatology, Kyorin University School of Medicine, Tokyo, Japan; 5 Department of Endocrinology, St. Luke’s International Hospital, Tokyo, Japan; 6 Center for Clinical Epidemiology, St. Luke’s International Hospital, Tokyo, Japan

**Keywords:** Rheumatic diseases, Thyroid diseases

## Abstract

We evaluated whether thyroid function test (TFT) screening is warranted for patients with autoimmune rheumatic diseases (ARD) by comparing the incidence of hypothyroidism requiring treatment (HRT) in ARD patients and healthy controls (HCs). Medical records of 2307 ARD patients and 78,251 HCs for whom thyroid-stimulating hormone (TSH) levels were measured between 2004 and 2018 were retrospectively reviewed. Cumulative incidence of HRT in ARD patients and HCs was compared. HRT development was evaluated with age- and sex-adjusted Kaplan–Meier curve. Risk factors were identified with Cox proportional hazard models. HRT was significantly more common in ARD patients than in HCs (6.3% vs. 1.9%, *P* < 0.001). After adjusting for age, sex, and baseline TSH level, hazard ratios for HRT were significantly higher in overall ARD patients (hazard ratio [95% confidence interval] 3.99 [3.27–4.87]; *P* < 0.001), particularly with rheumatoid arthritis and antinuclear antibody-associated diseases in female, and antinuclear antibody-associated diseases, spondyloarthritis, and vasculitis in male patients. Baseline high TSH level, thyroid-related autoantibody positivity, high IgG, and renal impairment were significant risk factors for hypothyroidism development in ARD patients; 20% of high-risk patients developed HRT during follow-up. HRT was significantly more frequent in ARD patients. Careful TFT screening and follow-up could help detecting clinically important hypothyroidism.

## Introduction

Hypothyroidism is a common comorbidity of autoimmune rheumatic diseases (ARD)^1,2^. Previous studies have reported that hypothyroidism is commonly associated with rheumatoid arthritis^3^, psoriatic arthritis^4^, antinuclear antibody-associated rheumatic diseases^5–8^, and other diseases including vasculitis^9–11^. Hypothyroidism manifests with musculoskeletal complaints, fatigue, constipation, edema, sicca, and mental symptoms^12^, which can be confused with the symptoms caused by ARD and glucocorticoid treatment.

Overt hypothyroidism is defined as a combination of high thyroid-stimulating hormone (TSH) with low free thyroxine (FT4) levels, whereas subclinical hypothyroidism is defined as a combination of high TSH with normal FT4 levels. A previous study has shown that subclinical thyroid dysfunction is associated with cardiovascular events and heart failure in the long term^13^. Treatment that includes thyroid hormone supplementation is required for clinical hypothyroidism, and subclinical hypothyroidism particularly with a TSH level of at least 10 µU/mL^14^.

Based on the high prevalence, thyroid function testing (TFT) is routinely performed at some rheumatology clinics, especially at initial assessment when ARD is suspected. In view of the high prevalence of hypothyroidism including the subclinical form without treatment indications, early recognition of hypothyroidism requiring treatment (HRT) is clinically meaningful to decide upon appropriate treatment. However, most previous studies have only analyzed the relationship between ARD and thyroid dysfunction, regardless of treatment indication. Moreover, previous studies included a limited number of patients, and detailed data on the frequency of HRT and its risk factors are lacking. Accordingly, the clinical usefulness of TFT screening in ARD patients has been controversial.

Therefore, we conducted this retrospective cohort study to evaluate and compare the cumulative incidence and risk factors of HRT in ARD patients and healthy controls (HCs).

## Methods

### Study participants

We retrospectively reviewed the medical records of patients with ARD who visited the Immuno-Rheumatology Center in St. Luke's International Hospital, Tertiary Care Center, Tokyo, Japan between January 2004 and July 2018. Diagnosis of ARD was based on a physician’s diagnosis registered in our medical records system. We included participants who had regular health checkups at the Center for Preventive Medicine in our institution during the same period as HCs. We included ARD patients or HCs who were followed up for at least 1 year and had their TSH levels measured at least once. We excluded patients below 18 years of age. We included only at-risk patients by excluding those with a history of thyroid dysfunction requiring treatment prior to the measurement of TSH levels at our institution. Thyroid dysfunction requiring treatment was defined as a condition with a TSH level of 10 µU/mL or more for hypothyroidism, or 0.1 µU/mL or less for hyperthyroidism as well as thyroid dysfunction for which a physician had initiated treatments (hormone supplementation treatment, levothyroxine, for hypothyroidism, and propylthiouracil, methyl-mercaptoimidazole and potassium iodide for hyperthyroidism).

### Data collection

We investigated the basic demographics and blood test results including complete blood count, chemistry, and thyroid function (TSH, free T4, and free T3) in both groups. In ARD patients, data regarding diagnosis of ARD, thyroid-associated antibodies such as anti-thyroid peroxidase (anti-TPO Ab) or anti-thyroglobulin antibodies (anti-Tg Ab), treatment for thyroid dysfunction and its date of initiation were identified. ARD was classified into rheumatoid arthritis, antinuclear antibody(ANA)-associated diseases (systemic lupus erythematosus, Sjogren syndrome, dermatomyositis, systemic sclerosis, and mixed connective tissue disease), spondyloarthritis (ankylosing spondylitis, psoriatic arthritis, reactive arthritis, inflammatory bowel disease-associated arthritis, and undifferentiated spondyloarthritis), vasculitis (antineutrophil cytoplasmic antibody (ANCA)-associated vasculitis, IgA vasculitis, cryoglobulinaemic vasculitis, Bechet disease, relapsing polychondritis, Goodpasture syndrome, polyarteritis nodosa, giant cell arteritis, and Takayasu disease), and other ARD (Polymyalgia rheumatica, remitting seronegative symmetrical synovitis with pitting edema syndrome, IgG4-related disease, and adult onset Still disease). In HCs, we collected data of a questionnaire which was used for a routine care at our preventive medicine center; all participants in health checkups were required to complete the questionnaire, which inquired about their medical history including details regarding thyroid dysfunction and treatment status.

### Outcome and definition of hypothyroidism with treatment indications

The primary outcome was the cumulative incidence of HRT during follow-up. We defined HRT as that with a TSH level of at least 10 µU/mL, or for which a physician had initiated hormone supplementation treatment. The secondary outcome included the proportion of patients with HRT detected at baseline assessment and in follow-up as well as the cumulative incidence of all hypothyroidism (TSH ≥ 4.5 µU/mL) during follow-up.

### Group classification based on a presence of ARD and baseline TSH level

We defined high baseline TSH as levels of ≥ 4.5 µU/mL at initial assessment based on the institutional reference range. Participants were divided into four groups: (1) HCs without high baseline TSH, (2) ARD patients without high baseline TSH, (3) HCs with high baseline TSH, and (4) ARD patients with high baseline TSH.

### Statistical analyses

The collected categorical data are presented as counts with percentages. Other data are presented as mean (SD) or median (IQR) for continuous variables of normal and non-normal distribution.

First, we described baseline characteristics of ARD patients and HCs. Thereafter we compared the cumulative incidence of HRT during the study period as well as the proportion of participants with HRT detected at the initial assessment between ARD and HCs using a Chi-square test.

Afterwards, to evaluate the necessity for monitoring TFT in participants who had no HRT at baseline assessment, a Kaplan–Meier curve and log-rank test was used to assess the differences in the development of HRT during follow-up. We defined the date of baseline assessment as the day when TSH was checked for the first time in our department in ARD patients or at the prevention medicine center in HCs. Patients were censored on the day of last visit, or on July 31, 2018 if they were followed over the study period. Participants were also censored if they developed hyperthyroidism with treatment indication. The hazard ratios (HR) for age, sex, ARD (and its type), and high baseline TSH were assessed using the Cox’s proportional hazards model as well as age- and sex-adjusted HR of groups 2–4 compared to group 1. The HR of ARD for HRT was also evaluated after stratifications by gender and quartiles of age in ARD patients. In addition, to identify ARD patients who should be carefully followed up, Cox’s proportional hazards model was performed to identify risk factors for the development of HRT.

We included variables with a *P* value < 0.1 in univariate analysis into multivariate analysis. A *P* value < 0.05 was considered significant for each analysis. All analyses were performed using R (version 3.3.2) and EZR, which is a graphical user interface for R^15^.

### Ethical approval

Informed consent was waived for retrospective review, with provisions for opting out. This study was approved by The St. Luke’s International University Institutional Review Board (number: 18-R093), with reference to relevant ethical guidelines for medical research in Japan.

## Results

### Baseline characteristics

We identified 2374 ARD patients and 79,369 controls, who were followed up for more than 1 year and had TSH levels measured at least once. A total of 75 (3.2%) patients and 1118 (1.4%) controls were excluded owing to a past medical history of thyroid dysfunction requiring treatment (hypothyroidism: 56 and 508, and hyperthyroidism: 15 and 655, respectively, in each group). A total of 2307 ARD patients and 78,351 controls were included in the analysis. The mean age (SD) of the ARD patients and controls was 53.7 (16.2) and 46.1 (11.9) years, respectively; 1826 (79.2%) and 38,632 (49.4%) of the ARD patients and controls, respectively, were women. A total of 446 (19.3%) ARD patients had multiple diseases, and about half (45.1%) of those patients had secondary Sjogren syndrome. The median follow-up period (days) was significantly longer in the control group (1365 [743, 2192] vs 1992 [958, 3632], *P* < 0.001). Other characteristics are summarized in Table 1.

**Table 1:** Baseline characteristic of autoimmune rheumatic disease patients and controls.

	Autoimmune rheumatic disease (*n* = 2307)	Control (*n* = 78,251)
Age (years)	53.7 (16.2)	46.1 (11.9)
Female (%)	1826 (79.2)	38,632 (49.4)
BMI (body mass index)	22.74 (3.9)	22.41 (3.3)
Follow-up (days)	1365 [743, 2192]	1992 [958, 3632]
**Blood test**		
Hemoglobin	12.1 (1.7)	13.9 (1.5)
Creatinine	0.57 [0.50, 0.68]	0.72 [0.61, 0.84]
Aspartate aminotransferase	18 [15, 21]	20 [17, 24]
Alanine transaminase	14 [11, 18]	18 [14, 26]
Creatinine kinase	57 [38, 85]	–
Low-density lipoprotein cholesterol	105 [87, 125]	115 [95, 136]
**Autoantibody**		
Rheumatoid factor positive (%)	961 (41.7)	6734 (8.6)
Cyclic citrullinated peptide positive (%)	588 (25.5)	–
Antinuclear antibody ≥ 80 (%)	813 (40.7)	–
Sjogren’s syndrome-A positive (%)	284 (12.3)	–
Sjogren’s syndrome-B positive (%)	79 (3.4)	–
**Autoimmune rheumatic disease**		
Rheumatoid arthritis (%)	1091 (47.3)	–
Spondyloarthritis (%)	161 (7.0)	–
Antinuclear antibody-associated disease (%)	944 (40.9)	–
Systemic lupus erythematosus (%)	363 (15.7)	–
Sjogren’s syndrome (%)	396 (17.2)	–
Polymyositis/dermatomyositis (%)	104 (4.5)	–
Systemic sclerosis (%)	222 (9.6)	–
Mixed connective tissue disease (%)	43 (1.9)	–
Vasculitis (%)	202 (8.8)	–
Others ARD (%)	244 (10.6)	–

Variables are shown as mean (*SD* standard deviation) or median (*IQR* interquartile range).

### Cumulative incidence of hypothyroidism with treatment indication in ARD patients and HCs

Results of TFT at baseline and during follow-up are summarized in Table 2. There were significant differences in the cumulative incidence of HRT, which was more frequent in ARD patients (146 [6.3%] vs. 1478 [1.9%]; *P* < 0.001). Hypothyroidism with treatment indications at the initial assessment was significantly more frequent in ARD patients than in controls (32 [1.4%] vs. 365 [0.5%]; *P* < 0.001). Most (129/146 [88.4%]) ARD patients with HRT received thyroid hormone supplementation.

**Table 2:** Results of thyroid function test and prevalence of hypothyroidism.

	Rheumatic disease (*n* = 2307)	Control (*n* = 78,251)	*P* value
Times of TSH measurement	2 [1, 5]	5 [3, 9]	< 0.001
Baseline TSH level (μU/mL)	1.76 [1.12, 2.69]	1.74 [1.20, 2.53]	0.953
< 4.5 μU/mL (%)	2115 (91.6)	73,835 (94.4)	< 0.001
≥ 4.5 μU/mL (%)	170 (7.4)	4051 (5.2)	< 0.001
≥ 10 μU/mL (%)	22 (1.0)	365 (0.5)	0.003
Baseline free T4 level (μU/mL)	1.15 [1.04, 1.28]	1.29 [1.18, 1.40]	< 0.001
**TSH level at follow-up**			
≥ 4.5 μU/mL (%)	378 (16.4)	11,099 (14.2)	0.003
≥ 10 μU/mL (%)	85 (3.7)	1228 (1.6)	< 0.001
Newly detected hypothyroidism with treatment indications (%)	146 (6.3)	1478 (1.9)	< 0.001
At baseline (%)	32 (1.4)	365 (0.5)	< 0.001
At follow-up (%)	114 (4.9)	1113 (1.4)	< 0.001
Treatment with hormone supplementation	129 (5.6)	718 (0.9)	< 0.001
Time to treatment (days)	204 [32, 952]	1133 [589, 2384]	< 0.001
Time to hypothyroidism requiring treatment (days)	204 [17, 1088]	797 [237, 2142]	< 0.001
**Thyroid-related autoantibodies**			
Positive anti-TPO Ab or anti-TG Ab (%)	253 (11.0)	–	NA
At baseline	130 (5.6)	–	NA
At follow-up (%)	123 (5.3)	–	NA

Variables are shown as mean (SD) or median [IQR].

*anti-TG Ab* anti-thyroglobulin antibody, *anti-TPO Ab* anti-thyroid peroxidase, *IQR* interquartile range, *SD* standard deviation, *T4* thyroxine, *TSH* thyroid-stimulating hormone.

### Risk of ARD for hypothyroidism with treatment indication during follow-up

Participants without HRT at baseline assessment were analyzed for evaluating the risk for developing the disease during follow-up. After adjusting for sex, age, and each other, overall ARD and baseline TSH level were significant risk factors (HR: 3.99 [3.27- 4.87]; *P* < 0.001, and 1.94 [1.90–1.98]; *P* < 0.001, respectively). There were different risks among ARD patients. Patients with rheumatoid arthritis and ANA-associated diseases were at a significantly high risk for HRT (HR 2.67 [1.95–3.65]; *P* < 0.001 and 3.45 [2.61–4.55]; *P* < 0.001) with adjustments for age, sex, and baseline TSH level. In ANA-associated diseases, those except for mixed-connect tissue disease were significant risks for HRT. On the other hand, spondyloarthritis, vasculitis and other ARD were not significant risk factors in multivariate analysis. (Table 3).

**Table 3:** Hazard ratio of autoimmune rheumatic diseases and baseline TSH for the development of hypothyroidism with treatment indication during follow-up.

	Crude HR [95% CI]	*P* value	Adjusted HR [95% CI]	*P* value
Age (years)	1.05 [1.04–1.05]	< 0.001	1.02 [1.02–1.03]	< 0.001
Female	1.51 [1.35–1.70]	< 0.001	1.32 [1.17–1.48]	< 0.001
Baseline TSH level (μU/mL)	1.98 [1.94–2.03]	< 0.001	1.94 [1.90–1.98]	< 0.001
Control	1 (reference)	–	1 (reference)	–
ARD patients^b^	5.42 [4.47–6.59]	< 0.001	3.99 [3.27–4.87]	< 0.001
Rheumatoid arthritis^b^	2.87 [2.10–3.93]	< 0.001	2.67 [1.95–3.65]	< 0.001
ANA-associated diseases^b^	4.23 [3.16–5.67]	< 0.001	3.45 [2.61–4.55]	< 0.001
Systemic lupus erythematosus^b^	2.35 [1.43–3.88]	< 0.001	2.62 [1.62–4.23]	< 0.001
Sjogren’s syndrome^b^	1.79 [1.10–2.91]	0.019	1.63 [1.01–2.62]	0.046
Polymyositis/dermatomyositis^b^	2.32 [0.94–5.70]	0.067	2.59 [1.06–6.34]	0.037
Systemic sclerosis^b^	3.53 [2.10–5.95]	< 0.001	2.73 [1.64–4.56]	< 0.001
Mixed connective tissue disease^b^	1.52 [0.46–4.98]	0.49	1.88 [0.56–6.35]	0.31
Spondyloarthritis^b^	1.95 [0.80–4.74]	0.14	1.61 [0.66–3.88]	0.29
Vasculitis^b^	2.06 [1.01–4.21]	0.048	1.54 [0.75–3.15]	0.24
Other diseases^b^	2.19 [1.15–4.17]	0.017	1.77 [0.93–3.37]	0.08
Group 1 (HCs without high baseline TSH)	1 (reference)	–	1 (reference)	–
Group 2 (ARD without high baseline TSH)^a^	7.48 [5.87–9.54]	< 0.001	5.66 [4.41–7.26]	< 0.001
Group 3 (HCs with high baseline TSH)^a^	23.2 [20.6–26.1]	< 0.001	20.1 [17.8–22.7]	< 0.001
Group 4 (ARD with high baseline TSH)^a^	73.3 [52.9–101.5]	< 0.001	50.2 [36.0–70.1]	< 0.001

*HR* hazard ratio, *CI* confidence interval, *TSH* thyroid-stimulating hormone.

^a^HR of each group was adjusted for age and sex in multivariate analysis.

^b^Adjusted for age, sex and baseline TSH level in multivariate analysis.

**Figure 1:**
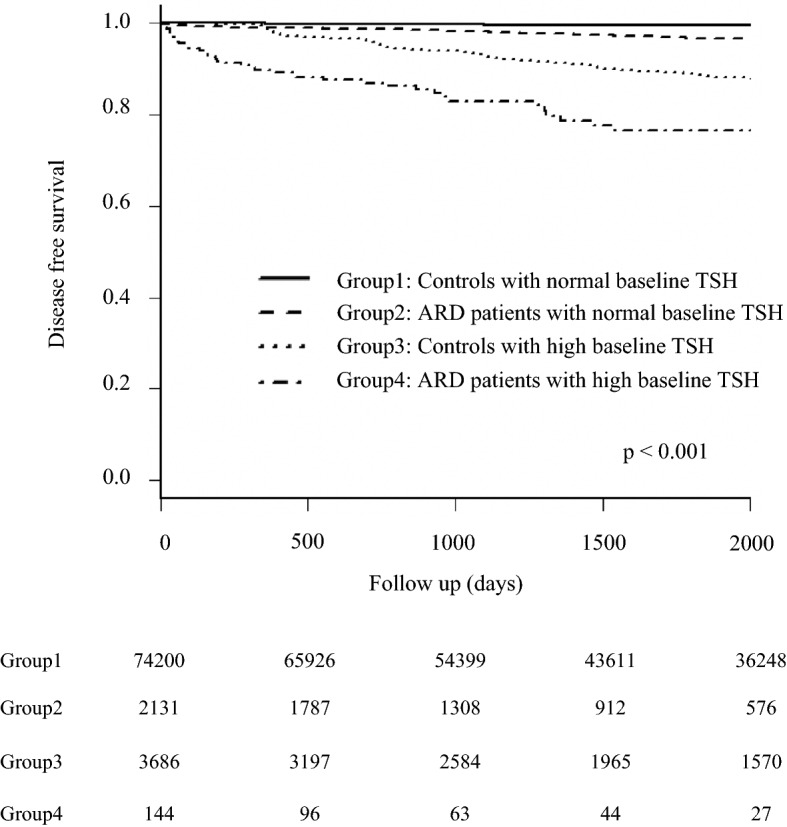
Age- and sex-adjusted disease-free survival of hypothyroidism requiring treatment in rheumatic disease patients and controls without treatment indication for hypothyroidism at baseline. *ARD* autoimmune rheumatic disease. *TSH* thyroid-stimulating hormone.

The age- and sex-adjusted Kaplan–Meier curve showed more frequent onset of thyroid dysfunction requiring treatment in groups 2–4 than in group 1 (Fig. 1). The HRs for HRT in groups 2–4 were significantly higher in Cox proportional hazard analysis (HR [95% CI] 5.66 [4.41–7.26] for group 2, 20.1 [17.8–22.7] for group 3, and 50.2 [36.0–70.1] for group 4 compared with group 1; *P* value < 0.001 in all instances) after adjusting for age (HR 1.02 [1.016–1.025]; *P* < 0.001) and female sex (HR 1.32 [1.17–1.48]; *P* < 0.001) (Table 3). Of 144 ARD patients who had high TSH level without treatment indication at baseline, 39 (27.1%) had HRT during follow-up.

### Risk of ARD for hypothyroidism with treatment indications stratified by age and sex

Considering differences in age and sex between patients with ARD and HCs, we stratified participants by sex and quartiles of ages in ARD patients. In a sex-stratified analysis, significantly higher HRs for HRT were observed in patients with ARD patients including rheumatoid arthritis, ANA-associated diseases, and other ARDs in female patients after adjusted for age and baseline TSH level. In contrast, ANA-associated diseases, spondyloarthritis, and vasculitis were significant risk factors for development of HRT during follow up in male participants (Table 4).

**Table 4:** Hazard ratio of autoimmune rheumatic diseases for hypothyroidism requiring treatment during follow-up stratified by sex.

	Female		Male	
	Adjusted HR	*P* value	Adjusted HR	*P* value
	[95% CI]		[95% CI]	
Age (years)	1.01 [1.00–1.02]	0.002	1.04 [1.03–1.04]	< 0.001
Baseline TSH level (μU/mL)	1.87 [1.82–1.92]	< 0.001	2.06 [1.99–2.13]	< 0.001
Control	1 (reference)	–	1 (reference)	–
ARD patients	4.40 [3.55–5.47]	< 0.001	2.65 [1.57–4.47]	< 0.001
Rheumatoid arthritis	3.15 [2.26–4.41]	< 0.001	1.34 [0.54–3.29]	0.53
ANA-associated diseases	3.12 [2.30–4.22]	< 0.001	4.80 [2.25–10.28]	< 0.001
Systemic lupus erythematosus	1.95 [1.16–3.27]	0.012	13.91 [3.03–63.80]	< 0.001
Sjogren’s syndrome	1.62 [0.98–2.68]	0.057	2.17 [0.46–10.28]	0.33
Polymyositis/dermatomyositis	2.93 [1.08–7.97]	0.035	0.96 [0.10–8.93]	0.97
Systemic sclerosis	2.71 [1.58–4.64]	< 0.001	5.52 [1.15–26.47]	0.033
Mixed connective tissue disease	2.01 [0.59–6.82]	0.26	No event	–
Spondyloarthritis	1.22 [0.39–3.81]	0.73	8.22 [2.01–33.62]	0.003
Vasculitis	1.38 [0.60–3.15]	0.45	6.25 [1.51–25.78]	0.011
Other diseases	2.49 [1.16–5.32]	0.019	1.10 [0.31–3.89]	0.88

*HR* hazard ratio, *CI* confidence interval, *TSH* thyroid-stimulating hormone, *ANA* antinuclear antibody.

When participants were stratified by sex and quartiles of age, overall ARD was a significant risk factor for HRT only in patients of 67 years old or older in males, whereas the risk of ARD for HRT was significantly higher consistently in any age group of female patients. Details were summarized in Table 5.

**Table 5:** Hazard ratio of autoimmune rheumatic disease for the development of hypothyroidism requiring treatment during follow-up stratified by age and sex.

Age (year)	< 42		42 ≦< 55		55 ≦< 67		67 ≦	
Gender	Female	Male	Female	Male	Female	Male	Female	Male
ARD patients, n	486	96	483	107	436	134	421	144
HCs, n	16,739	15,555	13,220	13,416	6595	8145	2078	2503
**Adjusted HR [95%CI]**								
HCs (reference)	1.0	1.0	1.0	1.0	1.0	1.0	1.0	1.0
Overall ARD^a^	4.29 [2.71–6.81]	No events	5.40 [3.63–8.03]	2.82 [0.70–11.47]	3.66 [2.22–6.04]	3.05 [0.75–12.44]	3.72 [2.41–5.76]	3.03 [1.61–5.71]
*P* value	< 0.001	–	< 0.001	0.15	< 0.001	0.12	< 0.001	< 0.001
Rheumatoid arthritis	3.47 [1.43–8.42]	No events	5.24 [3.08–8.91]	No events	2.16 [0.97–4.82]	1.86 [0.21–16.41]	2.02 [1.05–3.88]	1.56 [0.58–4.18]
*P* value	0.006	–	< 0.001	–	0.059	0.58	0.034	0.38
ANA-associated disease	2.28 [1.22–4.29]	No events	3.12 [1.72–5.67]	3.73 [0.83–16.74]	2.86 [1.39–5.86]	No events	3.73 [2.11–6.59]	6.53 [2.59–16.5]
*P* value	0.010	–	< 0.001	0.086	0.004	–	< 0.001	< 0.001
Spondyloarthritis	11.11 [2.55–48.5]	No events	3.24 [0.45–23.4]	No events	No events	8.30 [0.94–73.36]	No events	142.1 [12.65–1596]
*P* value	0.001	–	0.24	–	–	0.057	–	< 0.001
Vasculitis	6.48 [1.58–26.6]	No events	No events	2.19 [2.62–182.9]	8.34 [1.95–35.74]	No events	0.83 [0.19–3.60]	4.52 [0.61–33.62]
*P* value	0.010	–	–	0.004	0.004	–	0.80	0.14
Other diseases	2.19 [0.29–16.5]	No events	1.17 [0.15–8.85]	No events	3.59 [0.83–15.60]	No events	2.31 [0.71–7.52]	1.42 [0.40–4.95]
*P* value	0.45	–	0.88	–	0.088	–	0.16	0.59

Patients were stratified based on gender and quartiles of age of ARD patients.

*ARD* autoimmune rheumatic disease, *HR* hazard ratio, *CI* confidence interval, *ANA* antinuclear antibody.

^a^Adjusted for age and baseline thyroid-stimulating hormone level.

### Risk factors for hypothyroidism with treatment indications in ARD patients

To examine the characteristics of patients requiring careful thyroid function monitoring, we focused on ARD patients who did not have thyroid dysfunction requiring treatment at baseline. Cox proportional hazard models revealed that high baseline TSH, positive baseline anti-TPO Ab or anti-TG Ab, high IgG level (serum IgG ≥ 1700 mg/dL), and renal impairment (creatinine clearance ≤ 60 mL/min with the Cockcroft–Gault equation) were significant risk factors for the development of HRT during follow-up (Table 6).

**Table 6:** Risk factors for hypothyroidism requiring treatment during follow-up in ARD patients.

	Crude HR [95% CI]	*P* value	Adjusted HR [95% CI]	*P* value
Elderly (≥ 65 years)	1.77 [1.22–2.58]	0.003	1.11 [0.58–2.13]	0.762
Female	1.57 [0.91–2.70]	0.106		
Obesity (BMI ≥ 25 kg/m^2^)	1.02 [0.65–1.62]	0.925		
**Types of ARD**				
Rheumatoid arthritis	0.82 [0.57–1.19]	0.306		
ANA-Associated Disease	1.36 [0.94–1.96]	0.104		
Vasculitis	0.80 [0.39–1.63]	0.534		
Spondyloarthritis	0.64 [0.26–1.57]	0.329		
Other ARD	0.86 [0.45–1.65]	0.648		
**Blood test**				
Anemia (Hb ≤ 11 g/dL)	1.59 [1.07–2.35]	0.021	0.92 [0.53–1.61]	0.771
Renal impairment (Creatinine clearance ≤ 60 mL/min)	2.78 [1.69–4.57]	< 0.001	2.60 [1.21–5.60]	0.015
Liver dysfunction (AST ≥ 33 or ALT ≥ 39 IU/L)	1.59 [0.74–3.41]	0.237		
Dyslipidemia (LDL cholesterol ≥ 126 mg/dL)	0.94 [0.55–1.59]	0.808		
High IgG (≥ 1700 mg/dL)	1.79 [1.14–2.80]	0.011	1.89 [1.06–3.38]	0.031
Hypocomplementemia	1.46 [0.83–2.54]	0.188		
**Autoantibody**				
Rheumatoid factor positive	1.26 [0.85–1.88]	0.253		
Cyclic citrullinated peptide positive	1.04 [0.66–1.62]	0.877		
Antinuclear antibody ≥ 80	1.70 [1.15–2.49]	0.007	0.79 [0.45–1.40]	0.425
Sjogren’s syndrome-A positive	0.78 [0.42–1.46]	0.433		
**Thyroid condition**				
High thyroid-stimulating hormone (≥ 4.5 μU/mL)	9.29 [6.30–13.69]	< 0.001	2.83 [1.61–4.99]	< 0.001
Positive anti-thyroid peroxidase or anti-thyroglobulin antibodies	8.99 [6.22–12.99]	< 0.001	2.10 [1.24–3.54]	0.005

*HR* hazard ratio, *CI* confidence interval, *BMI* body mass index, *ANA* antinuclear antibody, *Hb* hemoglobin.

**Figure 2:**
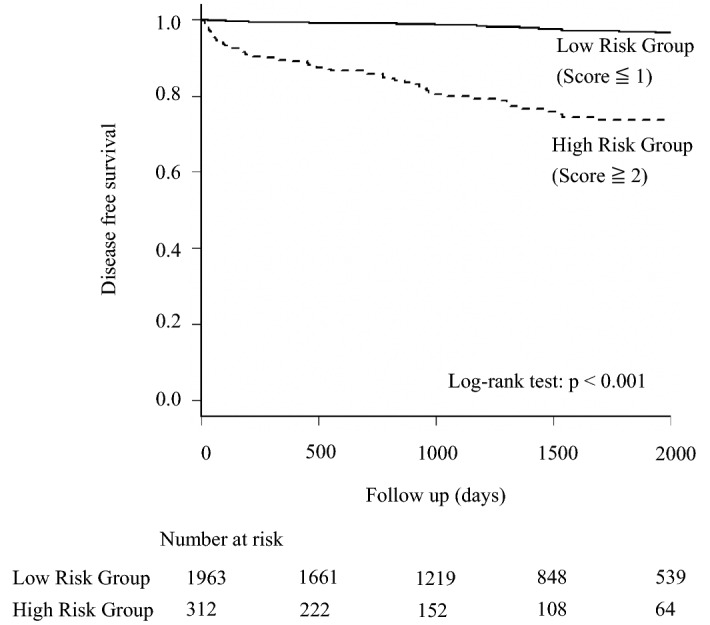
Disease-free survival of newly detected hypothyroidism requiring treatment in high-risk or low-risk patients with autoimmune rheumatic diseases.

Based on the coefficients acquired by the analysis, we assigned a score of one for positive baseline anti-TPO Ab or anti-TG Ab and high IgG, and a score of two for high baseline TSH and renal impairment. The receiver operating characteristic curve showed area under the curve of 0.779 [95% CI: 0.732–0.825]. When we defined patients with a score of 2 or more as a high-risk group, Kaplan–Meier curves and log-rank tests revealed a significant difference between high-risk (score ≥ 2) and low-risk (score ≤ 1) patients (*P* < 0.001) with respect to the onset of HRT (Fig. 2). The proportion of patients who developed HRT in low-risk and high-risk group were 14 (0.7%) and 32 (10.3%) at 1 year, 20 (1.0%) and 41 (13.1%) at 2 year, 24 (1.2%) and 53 (17.0%) at 3 year, 35 (1.8%) and 59 (18.9%) at 4 year, 40 (2.0%) and 62 (19.9%) at 5 year after initial assessment; *P* value < 0.001 for all.

### Risk factors for HRT in ARD patients with positive or negative anti-TPO Ab or anti-TG antibodies

Since anti-TPO or anti-TG antibodies were well known risk factors for hypothyroidism, we analyzed ARD patients with or without those antibodies separately. Of 546 ARD patients who checked these antibodies, there were 253 and 293 patients with or without those antibodies, respectively. Characteristics of patients were summarized in Supplementary Table S1. There were significantly more female patients, and those with Antinuclear antibody ≥ 80, ANA-associated disease in patients with positive anti-Tg and / or TPO antibodies. Newly detected HRT was significantly more frequent in patients with Anti-Tg and / or TPO antibodies (48 [16.4%] vs 74 [29.2%], *P* < 0.001) (Supplementary Table S1). In ARD patients with negative results for thyroid-related antibodies, baseline high TSH was an only significant risk factor for HRT. On the other hand, in ARD patients with positive those antibodies, high IgG level and baseline high TSH were identified as significant risk factors for HRT development, whereas ANA-associated disease was identified as a protective factor in this population (Supplementary Table S2).

## Discussion

Our study clearly revealed cumulative incidence of hypothyroidism with treatment indication was significantly higher in ARD patients when compared with HCs. Our study revealed that HRT was detected newly in 1.4% of ARD patients at initial assessment. In addition, cumulative incidence of hypothyroidism with treatment indications in ARD patients was 6.3%, which was significantly higher in ARD patients. The number increased to 202 (8.5%) of 2374 patients when those who already had a history of HRT before baseline assessment were included. The data of controls were compatible with those of a previous report, which indicated that the prevalence of overt hypothyroidism ranges between 0.3% and 1% in adults and may rise to 2% in elderly women^16^. Interestingly, despite slight differences in the number of patients solely with TSH ≥ 4.5 μU/mL, our study showed a large difference in the frequency of hypothyroidism with therapeutic indications.

We also demonstrated that patients with ARD overall had a significantly higher risk of hypothyroidism with treatment indication even after adjusted for confounding variables. In ARD, increased risk in rheumatoid arthritis and some of ANA-associated disease were significant, which was compatible with a previous report^5^. Our study revealed differences in risks of types of ARDs for HRT between male and female. In patients with rheumatoid arthritis, an increased risk for HRT was significantly increased only in female patients. In male patients, there were significantly increased risks for HRT in those with ANA-associated diseases, spondyloarthritis and vasculitis. The increased risk for HRT in patients with ANA-associated disease was consistent even after a stratification by sex. While we need to carefully interpret the significantly higher risk in male patients with spondyloarthritis and vasculitis considering a relatively small number and heterogeneity of diseases in these ARDs categories, the results were compatible with previous studies suggesting increased hypothyroidism in psoriatic arthritis^4^ or some vasculitis^9,11^. It is possible that there are different risks between male and female ARD patients since general frequency of hypothyroidism substantially differs between the sexes. In addition, it should be emphasized that we included only at-risk population for HRT. For instance, although coexistence of Sjogren’s syndrome and hypothyroidism is known, the HR of Sjogren’s syndrome for HRT development during follow up was not significant after stratification by sex. This was possibly due to the fact that Sjogren syndrome is often detected as a complication during the evaluation of patients newly diagnosed as having hypothyroidism, and such patients were excluded from this study.

Similar to previous studies, our study also revealed that a high baseline TSH level is a strong risk factor^17^. Careful follow-up of TFT should be recommended for ARD patients with high baseline TSH, because group 4 had a much higher risk as shown in Fig. 1. Furthermore, monitoring should be considered even in ARD patients with normal TSH at baseline, because group 2 had higher HR for HRT. We also identified risk factors for the onset of treatment-indicated hypothyroidism in ARD patients. High TSH level and positive anti-TPO Ab or anti-TG Ab were well known risk factors^17^. Patients with chronic thyroiditis or hypothyroidism were reported to have a significantly high IgG level^18^. Renal impairment is known to cause hypothyroidism with increased iodine concentrations consequent to low clearance of iodine^19^, particularly in patients with chronic thyroiditis who ingest sufficient or excessive iodine from foods^20^. In contrast to the relatively low cumulative incidence in low-risk patients, approximately 20% of high-risk patients will have hypothyroidism with treatment indications. We recommend that physicians should carefully monitor the thyroid function of high-risk ARD patients. High TSH level and renal impairment were risk factors for HRT in patients without anti-TPO or Tg antibody. Impressively, ANA-associated disease significantly reduced HRT development in ARD patients with anti-TPO and / or anti-Tg antibodies. This is probably because patients with ANA-associated disease more frequently have those antibodies, some of which are unrelated to clinically relevant hypothyroidism with treatment indication.

Besides high frequency, there are various reasons why detecting hypothyroidism is important for ARD patients. First, patients with hypothyroidism occasionally present with rheumatic symptoms^21^. Moreover, cardiovascular events are the leading cause of death in patients with chronic inflammatory diseases. Although chronic inflammation itself influences future cardiovascular events, some studies suggest that hypothyroidism and hypothyroidism-related hypercholesterolemia are responsible for coronary heart disease and cardiovascular death^13^ in patients with rheumatoid arthritis^22,23^. Several studies have shown that subclinical hypothyroidism may be associated with higher serum tumor necrosis factor-α levels and high disease activity in patients with rheumatoid arthritis^24,25^; poor response to treatments was also observed in patients with systemic lupus erythematosus with subclinical hypothyroidism^26^. These data suggested the importance of controlling thyroid function in ARD patients.

The efficacy and cost-effectiveness of thyroid function screening and follow-up in the general population have been much debated^27–29^. However, we speculate that thyroid function screening and follow-up in ARD patients, particularly in those with the above risk factors, are justified based on the result of this study.

This study includes several strengths. First, to the best of our knowledge, this is the largest study to assess the frequency of thyroid dysfunction requiring treatment in patients with various types of ARD. Our study also included a large number of controls whose thyroid function tests were regularly followed up. This allowed us to compare ARD patients and controls while providing detailed data on the level of TSH in the Japanese population. Nevertheless, this study has several limitations. First, this was a retrospective single-center study that had selection bias, and the decision to measure thyroid function and anti-TPO Ab or anti-Tg Ab in ARD patients depended on the judgment of each physician, whereas the TSH levels were measured in most (2378/2985; 79.7%) ARD patients and 80,439 (90.9%) of 88,450 HCs who were followed up for at least 1 year. Second, we did not obtain the data on medications that may affect thyroid function, such as amiodarone and contrast agents. Third, we did not consider central hyperthyroidism, transient increases in TSH, and non-thyroidal illness. However, a previous study has shown that a transient increase in TSH is common in patients with TSH levels of less than 7 μIU/mL; levels exceeding 10 μIU/mL are significantly associated with overt hypothyroidism^30^. Finally, although pregnant women were not excluded from this study, the prevalence of thyroid dysfunction requiring treatment may increase in view of the recommendations for more strict control of thyroid function in pregnancy^31^.

## Conclusions

We retrospectively compared the cumulative incidence of HRT and found that HRT was considerably more frequent in ARD patients. High TSH level, positive thyroid-related antibodies, high IgG and renal impairment were identified as significant risk factors in overall ARD patients. Therefore, we encourage routine active assessment of thyroid function in ARD patients, particularly in those with these risk factors. Further prospective studies are required to elucidate the natural history of thyroid dysfunction in ARD patients and to identify a suitable interval for thyroid function monitoring.

## Supplementary Information

**Supplementary Tables**.

## Data Availability

The data used in this study will be available on appropriate request to the corresponding author.
