# Supplementary material for: Clinical significance of monitoring hypothyroidism in patients with autoimmune rheumatic disease: a retrospective cohort study
Source: Sci Rep. 2021 Jul 5;11:13851. doi: 10.1038/s41598-021-93300-x (PMC8257694; doi:10.1038/s41598-021-93300-x)
Supplement: Supplementary file 1 — Supplementary Tables. [file 41598_2021_93300_MOESM1_ESM.docx]

**Supplementary material**

**Supplementary Table S1. Characteristics of ARD patients with or without Anti-Tg and / or anti-TPO antibody**

|  | Anti-Tg /TPO antibodies negative (n=293) | Anti-Tg / TPO antibodies positive (n=253) | p.value |
| --- | --- | --- | --- |
| Age (years) | 53.7 (16.5) | 51.8 (15.9) | 0.18 |
| Female (%) | 243 (82.9) | 230 (90.9) | 0.008 |
| Autoantibody |  |  |  |
| Rheumatoid factor positive (%) | 131 (52.8) | 113 (53.1) | 1 |
| Cyclic citrullinated peptide positive (%) | 59 (31.9) | 55 (30.6) | 0.82 |
| Antinuclear antibody ≥80 (%) | 115 (39.2) | 138 (54.5) | <0.001 |
| Sjogren’s syndrome-A positive (%) | 41 (16.5) | 41 (18.6) | 0.63 |
| Sjogren’s syndrome-B positive (%) | 12 (7.6) | 15 (10.5) | 0.42 |
| Autoimmune Rheumatic disease |  |  |  |
| Rheumatoid arthritis (%) | 123 (42.0) | 100 ( 39.5) | 0.60 |
| Spondyloarthritis (%) | 13 (4.4) | 8 (3.2) | 0.51 |
| ANA-associated disease (%) | 145 (49.5) | 148 (58.5) | 0.039 |
| Systemic lupus erythematosus (%) | 56 (19.1) | 54 (21.3) | 0.52 |
| Sjogren’s syndrome (%) | 62 (21.2) | 69 (27.3) | 0.11 |
| Polymyositis/dermatomyositis (%) | 18 (6.1) | 18 (7.1) | 0.73 |
| Systemic sclerosis (%) | 34 (11.6) | 35 (13.8) | 0.44 |
| Mixed connective tissue disease (%) | 10 (3.4) | 8 (3.2) | 1 |
| Vasculitis (%) | 23 (7.8) | 14 (5.5) | 0.31 |
| Others ARD (%) | 30 (10.2) | 25 (9.9) | 1 |
| Thyroid condition |  |  |  |
| Baseline TSH level (μU/mL) | 1.91 [1.11, 3.41] | 2.24 [1.31, 4.04] | 0.034 |
| TSH level at follow-up ≥4.5 μU/mL (%) | 97 (33.1) | 123 (48.6) | <0.001 |
| TSH level at follow-up ≥10 μU/mL (%) | 27 (9.2) | 44 (17.4) | 0.005 |
| Newly detected hypothyroidism with treatment indications (%) | 48 (16.4) | 74 (29.2) | <0.001 |
| At baseline (%) | 11 (3.8) | 18 (7.1) | 0.088 |
| At follow up (%) | 37 (12.6) | 56 (22.1) | <0.001 |
| Treatment with hormone supplementation | 35 (11.9) | 74 (29.2) | <0.001 |

ARD, autoimmune rheumatic disease; Tg, thyroglobulin; TPO, thyroid peroxidase; TSH, thyroid stimulating hormone; ANA, Antinuclear antibody

**Supplementary Table S2. Risk factors for HRT in ARD patients with or without anti-Tg /TPO antibody**

|  | Without Anti-TPO or Tg antibody (n=293) | | With Anti-TPO or Tg antibody (n=253) | |  |
| --- | --- | --- | --- | --- | --- |
|  | Crude HR | P value | Crude HR | P value | |
|  | [95% CI] |  | [95% CI] |  |  |
| Elderly (≥ 65 years) | 2.99 [1.56-5.71] | < 0.001 | 1.50 [0.84-2.68] | 0.18 | |
| Female | 0.51 [0.23-1.16] | 0.091 | 2.15 [0.52-8.83] | 0.29 | |
| Types of ARD |  |  |  |  | |
| Rheumatoid arthritis | 1.06 [0.55-2.04] | 0.86 | 1.46 [0.86-2.47] | 0.16 | |
| ANA-Associated Disease | 0.90 [0.47-1.72] | 0.76 | 0.54 [0.32-0.92] | 0.022 | |
| Vasculitis | 0.29 [0.04-2.17] | 0.23 | 1.08 [0.34-3.45] | 0.90 | |
| Spondyloarthritis | 1.53 [0.37-6.35] | 0.56 | 1.06 [0.26-4.36] | 0.93 | |
| Other ARD | 1.57 [0.61-4.07] | 0.35 | 0.43 [0.10-1.77] | 0.24 | |
| Blood test |  |  |  |  | |
| Anemia (Hb ≤ 11 g/dL) | 1.66 [0.84-3.25] | 0.14 | 1.16 [0.63-2.12] | 0.64 | |
| Renal impairment  　(Creatinine clearance ≤ 60 mL/min) | 3.38 [1.57-7.30] | 0.002 | 2.31 [0.98-5.43] | 0.056 | |
| Liver dysfunction | 1.66 [0.40-6.91] | 0.49 | 1.42 [0.51-3.93] | 0.5 | |
| (AST ≥ 33 or ALT ≥ 39 IU/L) |  |  |  |  | |
| Dyslipidemia | 1.82 [0.75-4.39] | 0.19 | 0.91 [0.42-1.98] | 0.81 | |
| (LDL cholesterol ≥ 126 mg/dL) |  |  |  |  | |
| High IgG (≥ 1,700 mg/dL) | 1.63 [0.72-3.68] | 0.24 | 1.98 [1.06-3.69] | 0.032 | |
| Hypocomplementemia | 0.77 [0.27-2.16] | 0.61 | 1.02 [0.48-2.15] | 0.96 | |
| Autoantibody |  |  |  |  | |
| Rheumatoid factor positive | 1.04 [0.52-2.08] | 0.92 | 1.21 [0.68-2.13] | 0.52 | |
| Cyclic citrullinated peptide positive | 1.55 [0.68-3.54] | 0.30 | 1.62 [0.88-2.98] | 0.12 | |
| Antinuclear antibody ≥ 80 | 1.13 [0.59-2.16] | 0.71 | 0.77 [0.45-1.31] | 0.33 | |
| Sjogren’s syndrome-A positive | 0.55 [0.17-1.80] | 0.32 | 0.46 [0.20-1.08] | 0.075 | |
| Thyroid condition |  |  |  |  | |
| High thyroid-stimulating hormone (≥4.5 μU/mL) | 5.91 [3.04-11.5] | < 0.001 | 3.16 [1.82-5.47] | <0.001 | |
|  | Adjusted HR | P value | Adjusted HR | P value | |
|  | [95% CI] |  | [95% CI] |  |  |
| Elderly (≥ 65 years) | 1.72 [0.78-3.78] | 0.18 |  |  | |
| Female | 0.58 [0.24-1.38] | 0.22 |  |  | |
| ANA-associated Disease |  |  | 0.44 [0.20-0.96] | 0.040 | |
| Renal impairment  (Creatinine clearance ≤ 60 mL/min) | 2.23 [0.93-5.34] | 0.072 | 2.42 [0.81-7.15] | 0.11 | |
| High IgG (≥ 1,700 mg/dL) |  |  | 2.57 [1.17-5.65] | 0.019 | |
| Sjogren’s syndrome-A positive |  |  | 0.69 [0.24-1.97] | 0.49 | |
| High thyroid-stimulating hormone (≥4.5 μU/mL) | 4.50 [2.19-9.22] | < 0.001 | 2.28 [1.03-5.03] | 0.041 | |

ARD, autoimmune rheumatic disease; Tg, thyroglobulin; TPO, thyroid peroxidase; HR, hazard ratio; CI, confidence interval; ANA, Antinuclear antibody; TSH, thyroid stimulating hormone; Hb, hemoglobin
